# Pilot Test of Aprendiendo Juntos/Learning Together Demonstrates Improved Self-Efficacy for Providing Care Among Latino Family Caregivers of Persons Living With Dementia

**DOI:** 10.1177/01939459251359209

**Published:** 2025-08-04

**Authors:** Carole L. White, Byeong Y. Choi, Roxana E. Delgado, Daria B. Neidre, Kimberly S. Peacock, Luis P. Luy, Roman Fernandez, Fayron Epps, Lixin Song

**Affiliations:** 1 School of Nursing, UT Health San Antonio, San Antonio, TX, USA; 2 Department of Population Health Sciences, UT Health San Antonio, San Antonio, TX, USA

**Keywords:** family caregivers, self-efficacy, Latino or Hispanic, dementia, intervention, randomized controlled trial

## Abstract

**Background:**

Few interventions have been culturally and linguistically adapted to address Latino family caregivers’ unique needs for support in providing dementia care.

**Objective:**

We sought to pilot test the Aprendiendo Juntos/Learning Together intervention in increasing self-efficacy for care provision among Latino caregivers.

**Methods:**

Using a randomized waitlist controlled design, 50 Latino family caregivers were assigned to either the immediate intervention group (IIG) or waitlist control group (WLG). The intervention was a 6-week psychoeducational program, delivered weekly via a video-conferencing platform, addressing topics around care provision such as communication, home safety, and medication management. Caregivers were assessed at baseline, 12, and 24 weeks. The primary outcome was caregiver self-efficacy at 12 weeks, with secondary outcomes including caregiver confidence, global health, and appraisal of behavioral symptoms of dementia. Between-group differences were examined using independent *t*-tests and multivariable linear regression, controlling for potential confounders.

**Results:**

Caregiver self-efficacy significantly improved in the IIG compared with the WLG (*P* = .042) with a moderate effect size (*d* = 0.64). Confidence in providing complex care also increased significantly in the IIG (*P* = .002), demonstrating a strong effect size (*d* = 1.00). In addition, 2 of the 4 subscales of the Caregiver Confidence in Medical Sign/Symptom Management scale—managing cognitive signs/symptoms and general medical management/responsiveness—showed significant improvements (*d* = 0.95, *P* = .004 and *d* = 0.99, *P* = .003, respectively). Sustainability of intervention effects at 24 weeks was observed in the IIG.

**Conclusions:**

The findings support the efficacy of the intervention in increasing self-efficacy in providing complex care to their family members with dementia among Latino caregivers.

More than 80% of the assistance needed by older adults is provided by family members or friends, with nearly half of these caregivers providing this care to someone with Alzheimer’s disease or another form of dementia.^1^ Within the United States, there are over 11 million family members/friends caring for a person living with dementia.^1^ Dementia caregiving is a demanding job that can last for many years with physical, emotional, and financial consequences for the caregiver. Although dementia caregivers report positive feelings about caregiving, they frequently report higher levels of burden and emotional distress compared with those caring for persons with other conditions or non-caregivers.^1^

Latino family caregivers are particularly impacted by dementia, not only related to the high prevalence of caregiving among Latino families, but also to the disproportionate impact on their health.^2^ While they are more likely to provide more intense caregiving in terms of hours and level of care than other populations, Latino caregivers are less likely to use community support and formal care services.^2^ Not surprisingly, poorer health outcomes are reported among Latino caregivers compared with non-Latino caregivers, including self-rated health and depression.^3,4^

## Caregivers Provide Complex Care

Family caregivers provide, on average, nearly 31 hours of care per week for their family members living with dementia.^1^ This care is defined as complex related to the nature of the care required by a person living with dementia. In addition to assisting with personal care and numerous instrumental activities of daily living, caregivers coordinate care among multiple healthcare providers and services. Caregivers also take on medical/nursing activities such as monitoring complex medication regimens and managing assistive devices for mobility, previously under the purview of a healthcare professional.^5^ All of these tasks involve the caregiver knowing when the task is needed, how to perform the task, understanding potential side effects to look for, and knowing when to call for help.^5^ Providing care to a person living with dementia becomes much more complex, related to communication difficulties the person may be experiencing as well as dementia-related behaviors. For example, persons living with dementia may be unable to communicate their pain, so the caregiver requires specialized knowledge to assess for pain in this context, to decide how and when to intervene, and to communicate effectively about the pain to the healthcare provider. Disturbances in behavior further complicate care provision when the care recipient may not understand the care task, become fearful, and resist care.^6^

Caregivers report limited support in learning how to provide this complex care, with their lack of preparation compounding their stress and anxiety.^5,7,8^ Caregivers require specialized knowledge to increase their competency and enable them to confidently provide quality care for persons living with dementia. Targeting self-efficacy, the belief in one’s ability to execute behaviors to achieve desired outcomes, may enhance the caregiver’s ability to provide care and, in turn, improve caregiver outcomes. The results of an integrative review identified the prevalence of complex care tasks performed by family caregivers of persons living with dementia, including managing multiple medications, wound care, and nutritional management.^9^ Caregivers describe their lack of confidence in undertaking these tasks, which results in anxiety, worry, stress, and guilt. Low self-efficacy has been associated with negative caregiver health outcomes, including burden and depression.^10^ Thus, strategies to improve caregiver self-efficacy should be an integral component of caregiver interventions.

It should be noted that several studies have reported greater self-efficacy for caregiving among Latino family caregivers compared with non-Latino White caregivers^11,12^ and a negative association between self-efficacy and caregiver burden and depression among Latino caregivers residing in the United States.^13^ It has been suggested that their self-efficacy may be linked to their internalized beliefs about their role in providing care for their family and thus impact how they perceive their caregiving experience.^14^ Losada-Baltar et al^13^ reported higher self-efficacy among Latino caregivers who had greater family support. Beliefs about their family responsibility for care could, however, lead caregivers to be reluctant to access formal services and to prioritize the needs of their recipient of care and neglect their own self-care, contributing to the adverse health outcomes observed among Latino caregivers. This suggests the importance of a focus on strategies for self-care, including family support, in interventions aimed at building self-efficacy among Latino caregivers.

## Building Self-Efficacy for Complex Care

To address a gap in evidence-based psychoeducational programs targeting caregiver self-efficacy in providing complex care to persons living with dementia, we created Learning Skills Together (LST). The intervention, developed by an interprofessional healthcare team, was based on principles of adult education with Bandura’s theory of self-efficacy guiding the design and evaluation of LST.^15^ The intervention was initially delivered to 171 caregivers as part of the service program for family caregivers provided by a School of Nursing. Participating caregivers reported high satisfaction and strongly agreed that the intervention provided them with useful and important information to support their caregiving role.^16^ Based on these encouraging results, a pilot study was conducted to formally evaluate LST using a one-group pre–post-intervention design (n = 35). The intervention demonstrated significant improvements in overall caregiver competence and self-efficacy in providing complex care.^17^

There are limited Spanish-language materials and few that have been culturally adapted to support Latino family caregivers.^1^ There is an urgent need to develop culturally appropriate interventions to provide education and support for the growing number of Latino family caregivers.

## Purpose

The gap in culturally accessible support for Latino caregivers, coupled with the positive findings from the pilot testing of the LST intervention, motivated the current study. To address the unique needs of Latino family caregivers, a participatory research approach was used to create Aprendiendo Juntos/Learning Together, a culturally and linguistically adapted version of LST. The current study is a stage I/II study based on the National Institutes of Health Stage Model for Behavioral Intervention Development.^18^ The purpose of this pilot study was to examine the efficacy of the Aprendiendo Juntos/Learning Together bilingual intervention in building self-efficacy for care provision among Latino caregivers of people living with dementia.

## Methods

### Design

This pilot study utilized a longitudinal, randomized, waitlist controlled trial to compare caregiver self-efficacy, the primary outcome, between the immediate intervention group (IIG) and the waitlist control group (WLG). Approval for the study was received from the Institutional Review Board of the University of Texas Health Sciences Center at San Antonio (HSC 22-579H). All participants provided written informed consent prior to participation in the study. The study was guided by a Latino Family Caregiver Council, consisting of members of the research team and 5 Latino family caregivers.

### Participants and Recruitment

Latino family caregivers were primarily recruited through an integrated service and research program for families living with dementia that is housed in a School of Nursing. A community engagement program in South Texas aimed at identifying research priorities for dementia care among Latino families, as well as members of the Latino Family Caregiver Council, supported recruitment activities. Recruitment activities were concurrent with the conduct of the study, taking place between September 2022 and November 2023.

**Figure 1.**
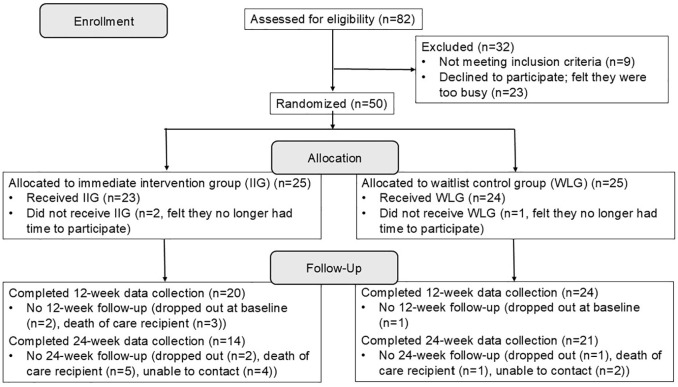
Construction of the study sample.

Eligible caregivers self-identified as a Latino (Hispanic) primary or main caregiver to a family member/friend diagnosed with some form of dementia and were aged 18 years or older. They reported assisting their recipient of care with at least 2 instrumental activities of daily living or 1 activity of daily living. Caregivers who were unable to speak and read either English or Spanish were excluded. Participants were asked to have reliable access to the internet and email and to commit to attend synchronous sessions using the Zoom videoconferencing platform. To be more inclusive in our recruitment, we were able to provide tablets and internet access as well as training on Zoom to caregivers who did not have the required technology. Of the potential participants identified through recruitment activities, 82 were screened for eligibility. Of those, 32 were excluded or declined to participate, the most common reasons being that they felt they were too busy or the class schedule was not convenient related to other commitments (Figure 1).

### Procedures

The study team was trained in all study procedures by the primary investigator. Personnel facilitating the intervention had previous experience working with family caregivers and in group facilitation. They were provided with preparatory materials to review and participated in a workshop that reviewed the intervention content and best practices for the facilitation. The Research Electronic Capture data management system, a secure online system, was utilized for the study, including electronic informed consent, randomization, and data collection.

Potential participants completed the screening questionnaire by telephone and, if eligible, were sent the electronic consent form for their review. They had an opportunity to discuss the study with a member of the study team, have their questions answered, and if wishing to proceed, provided written consent. To ensure a minimum of 3 caregivers per intervention group, we recruited cohorts of at least 6 caregivers. Once a cohort had been recruited and within 2 weeks of conducting the intervention, consented caregivers were randomized in a 1:1 allocation, using variable block sizes of 2, 4, and 6, to the IIG or to the WLG. Following randomization, baseline data were collected on all participants. Printed program binders were mailed to the caregivers randomly assigned to the IIG. The Aprendiendo Juntos/Learning Together intervention was then delivered via Zoom weekly for 6 weeks to the caregivers in the IIG. At 12 weeks (6 weeks after the IIG completed the intervention), follow-up data were collected from caregivers in both the IIG and WLG. Printed binders were then mailed to the WLG, who crossed over to the intervention arm and received the Aprendiendo Juntos/Learning Together intervention. The intervention was delivered in both English and Spanish. Follow-up data were collected at 24 weeks (6 weeks after the WLG received the intervention).

Data were collected via Zoom or telephone, in English or Spanish, depending on caregiver preference, by research assistants. Baseline data were collected most frequently via Zoom to ensure caregiver comfort with the technology. Research assistants collecting follow-up data were, for the most part, blinded to group assignment, although there were several instances related to the small research team where a research assistant assisted with the intervention delivery and collected follow-up data.

### Aprendiendo Juntos/Learning Together Intervention

Self-efficacy theory underpins the Aprendiendo Juntos/Learning Together psychoeducational bilingual intervention. Various strategies, hypothesized by Bandura to build self-efficacy, including mastery experiences, vicarious experiences, verbal persuasion, and physiological and affective states, are integrated into each weekly session.^15^

The selection of topics included in the intervention was guided by findings from a seminal report that identified the complex care tasks that caregivers reported performing most frequently and/or the tasks that they reported as difficult.^5,19^ The Latino Family Caregiver Council confirmed the importance of the topics to Latino family caregivers and also their recommended sequence. Topics included behaviors that challenge and strategies to support the care dyad, approaches to optimize communication with the person living with dementia, managing medications, and supporting safe mobility and transfers. The Council particularly emphasized the importance of a focus on self-care throughout the intervention, suggesting that the cultural emphasis on familism may lead caregivers to neglect their self-care.^20^ They also recommended a discussion early in the intervention on building a care team that can support the caregiver. The expansion on self-care, including a discussion about a care team early in the intervention, as well as increased content on understanding dementia and its progression, were added to the original design of the intervention. Further information on the cultural tailoring of the intervention is being published separately (manuscript under review).

The specific strategies utilized in delivering the intervention were based on the sources of self-efficacy. For example, videos were recorded with caregivers describing strategies they had found helpful in communicating with their family members. These were used to tap into 2 sources of self-efficacy, verbal persuasion, and vicarious experiences. A practice exercise such as practicing oral care with their family member was integrated into the weekly homework, with the intent to build their mastery experiences. Table 1 presents the full list of topics along with key strategies targeting sources of self-efficacy.

**Table 1.** Intervention Topics and Strategies Used to Target Sources of Self-Efficacy.

Week	Content
Week 1	Overview of dementia; values underlying caregiving; building your care team; caring for yourself while caring for someone with dementia
Week 2	Behaviors that challenge—what are they, why do they occur, and how to intervene; supporting communication with someone with dementia
Week 3	Home safety; supporting safe mobility and safe transfers
Week 4	Strategies to support eating and drinking; identifying swallowing difficulties and ways to support safe nutrition; managing oral care
Week 5	Safety around medications; urinary incontinence; recognizing signs of urinary tract infections and ways to prevent infections
Week 6	Pain assessment; planning for the future—navigating the changing needs of your family member, including transition to higher levels of care, palliative and hospice care
Targeted source of self-efficacy	Key strategies
Physiological and affective states	Nurturing environment where the experiences of all caregivers are valued
Verbal persuasion	Expert healthcare practitioners providing the intervention content
Mastery experiences	Practice exercises where caregivers are able to achieve weekly goals around different aspects of care provision and self-care
Vicarious experiences	Opportunities for caregivers to hear other caregivers describing their successes and their challenges
Verbal persuasion	Positive feedback from the intervention team about caregiver achievements while viewing their challenges as opportunities for growth and improvement
Mastery experiences; Vicarious experiences	Storytelling where caregivers described their experiences and information about resources and things they had found helpful, thus building their sense of mastery
Vicarious experiences	Case studies that engaged caregivers in discussion and helped them to visualize success
Vicarious experiences; verbal persuasion	Role modeling from caregivers through the videos recorded with caregiver peers
Physiological and affective states; vicarious experiences; mastery experiences	Focus on self-care embedded in each weekly session to support caregivers in managing their stress, and tips shared among caregivers for maintaining a healthy lifestyle

The intervention was delivered weekly for 6 weeks, in English or Spanish, via a video-conferencing platform. During each 1.5-hour interactive session, different topics around dementia care provision were addressed. Caregivers followed the content in their program binder, used as a structure and support for each session. Intervention materials were available in both English and Spanish. Each topic was introduced with a short (<10 minutes) video-recorded presentation by a healthcare professional with expertise in the specific topic. There were also recorded discussions with caregivers around certain topics. For example, the content on addressing communication challenges was enhanced with a discussion between 2 caregivers and a speech-language pathologist on how to address word-finding difficulties. Caregivers were provided with essential questions that they were asked to reflect on during the presentations, and which also served to guide the discussion. Case studies were utilized to reinforce the content and to engage the caregivers in discussion, with caregiver storytelling prioritized. Caregivers were encouraged to discuss their personal situation as it related to the content and to share helpful tips and resources with the group.

During the week following the session, caregivers were instructed to reflect on the essential questions and how the content specific to these questions could be applied to their personal caregiving circumstances. A practice activity was embedded in a question, designed to reinforce the content from that week. For example, a home safety guide was provided during the presentation on home safety. Caregivers were instructed to use this guide during the following week to identify changes they may need to make to their home to improve its safety and to make it easier for them to provide care. One of the essential questions from that week was to consider, among the changes they may need to make, what changes they would prioritize. The following week, caregivers were asked to share their process of applying the content to their caregiving situation, challenges they may have experienced, and benefits they may have noted.

The research coordinator, 2 nurses, and 2 researchers participated in the weekly sessions. A research assistant provided support for the technical aspects of the sessions, including assisting any caregiver who may have had difficulties with Zoom.

### Measures

The measures included primary and secondary outcomes collected at baseline upon enrollment, and at 12 and 24 weeks post-baseline. Participants’ demographics and other confounding variables were collected at baseline.

The primary outcome was overall self-efficacy, measured using the 8-item Caregiver Self-Efficacy Scale (CSES-8), developed and validated in both English and Spanish.^21,22^ Scores range from 1 to 10, with higher scores indicating higher self-efficacy. In 2 samples of family caregivers, the 8-item scale showed good internal consistency (Cronbach’s α = 0.89 and 0.88) and evidence supporting construct validity.^22^ In this study sample, Cronbach’s alpha was 0.70.

To further examine self-efficacy, we included 2 scales that more specifically examine caregiver confidence around different aspects of care provision, including confidence to perform specific tasks as well as managing medical signs/symptoms. The Complex Care Task Scale, a 15-item unidimensional scale developed by the study team in a previous study, was used to assess caregiver confidence with various complex care tasks.^17^ Scores range from 1 to 5, with higher scores demonstrating higher levels of confidence in performing complex care tasks. The questionnaire has shown high internal consistency in the previous and the current studies (α = 0.89 and α = 0.85 respectively). The Caregiver Confidence in Sign/Symptom Management Scale, with subscales for knowledge about signs/symptoms (α = 0.56), managing cognitive signs/symptoms (α = 0.82), managing medical signs/symptoms (α = 0.78), and general medical management/responsiveness (α = 0.94), was also administered.^23^ Each sub-scale is scored from 1 to 5, with higher scores indicating greater confidence. Internal consistency in the current sample was high, with Cronbach’s alphas ranging from 0.87 to 0.95.

Secondary outcomes included global health and appraisal of behavioral symptoms of dementia. The PROMIS Global Health V1.2 was used to assess the caregivers’ general perceptions of their physical and mental health.^24^ Appraisal of behavioral symptoms of dementia was assessed with the Revised Memory and Behavior Checklist, which asks caregivers about the presence, during the past week, of 24 behaviors that may occur among people living with dementia, and if present, their appraisal of the behavior.^25^ The number of times the care recipient was hospitalized and/or went to the emergency room within the previous 3 months was also collected.

Caregiver demographics and information about caregiving (eg, kin relationship to care recipient, years of caregiving), as well as scales measuring potential confounders, were collected at baseline. Social support was measured using the abbreviated Lubben Social Network Scale, which demonstrates adequate internal consistency (α = 0.83), as well as discriminant and convergent validity across cultures.^26^ Health literacy was assessed using the Short Assessment of Health Literacy, an 18-item scale that strongly correlates with other validated health literacy scales (*r* = 0.94) and is highly reliable (α = 0.89).^27^ To measure the number of complex care tasks performed by family members, we used a count-based assessment with 17 items where caregivers indicate if they perform the task and if so, alone or with assistance.^5^

Measures were administered in English or Spanish, depending on caregiver preference. For those scales that did not have a validated Spanish version, forward and backward translation was conducted by the study team, with the Spanish version of the scales reviewed by the Spanish-speaking caregivers participating in the Latino Family Caregiver Council.

### Data Analysis

A sample size of 48 (24 per group) was calculated, assuming a moderate effect size, a type I error of .05 and a type II error of .20 (80% power). In the LST study,^17^ we observed an attrition rate of 15%, so we aimed to recruit 56 participants.

Frequencies (percentages) and means (SDs) are presented for categorical and numerical descriptive statistics, respectively. The primary endpoint for analysis was the change in self-efficacy at 12 weeks, 6 weeks after the end of the intervention for the IIG and before the WLG crossed over to participate in the intervention. Between-group differences were examined using independent *t*-tests. Effect sizes of the differences were calculated. Given the sample size, it was unlikely that all covariates between the groups would be completely balanced; multivariable linear regression was used to examine the impact of the intervention on the outcomes while adjusting for potential confounders. Particularly, the intervention was provided in both Spanish and English, and therefore, language was included as a covariate in the models.

We examined the 24-week data from the IIG only (18 weeks after they had completed the intervention) to examine for sustainability of the intervention effects. We compared confidence outcomes between baseline and 12 weeks, baseline and 24 weeks, and 12 and 24 weeks using paired t-tests. We hypothesized that changes in confidence outcomes would be statistically significant between baseline and 12 weeks, baseline and 24 weeks, but not between 12 and 24 weeks, thereby suggesting sustainability of the intervention effects. A 2-sided *P*-value <.05 was used for statistical significance. Analyses were performed in R version 4.3.2 (R Foundation for Statistical Computing, Vienna, Austria).

## Results

### Participant Characteristics

The study sample consisted of 50 Latino caregivers recruited from across North America, including Puerto Rico, with the majority from South Texas (84%). Table 2 presents baseline characteristics stratified by intervention group. Of note, most caregivers (92%) were female, the mean age was 59 years, and the majority were adult children caring for a parent (72%). Groups were similar in demographic and caregiving characteristics although those in the WLG were more likely to be employed than those in the IIG (40% vs 20%, respectively) and report caregiving for >5 years (56% vs 36%). Randomization was not stratified by language (English vs Spanish), with a higher number of caregivers who preferred English in the IIG (76%) compared with the WLG (56%).

**Table 2.** Baseline Demographic and Caregiving Characteristics by Intervention Group.

Variable	IIG (n = 25)	WLG (n = 25)
Gender, n (%)		
Female	23 (92%)	23 (92%)
Male	2 (8%)	2 (8%)
Age, mean ± SD, years	61 ± 11	57 ± 11
Preferred language for intervention, n (%)		
English	19 (76%)	14 (56%)
Spanish	6 (24%)	11 (44%)
Hispanic, Latino, or Spanish origin, n (%)		
Mexican, Mexican American, Chicano	17 (68%)	20 (80%)
Puerto Rican	5 (20%)	5 (20%)
Cuban	2 (8%)	0 (0%)
Other	1 (4%)	0 (0%)
Education level, n (%)		
Secondary level/GED equivalent	2 (8%)	3 (12%)
Some college/associate’s degree/vocational training	7 (28%)	6 (24%)
Bachelor’s degree or higher, n (%)	16 (64%)	16 (64%)
Sufficient money to meet basic living needs, n (%)		
Always enough money	20 (80%)	19 (76%)
Some of the time/never	5 (20%)	6 (24%)
Changed employment status to provide care, n (%)	16 (64%)	15 (60%)
Employed full time or part time, n (%)	5 (20%)	10 (40%)
Current marital status, n (%)		
Married/common law	18 (72%)	17 (68%)
Divorced/separated/widowed	5 (20%)	3 (12%)
Never married	2 (8%)	5 (20%)
Kin relationship to recipient of care, n (%)		
Spouse	4 (16%)	3 (12%)
Parent	18 (72%)	18 (72%)
Other	3 (12%)	4 (16%)
Time caregiving, n (%)		
<1 year	3 (12%)	0 (0%)
1 to 3 years	7 (28%)	8 (32%)
>3 to 5 years	6 (24%)	3 (12%)
>5 years	9 (36%)	14 (56%)
Previously provided care to a relative, n (%)	16 (64%)	13 (52%)
Experience working in the healthcare field, n (%)	9 (36%)	7 (28%)
Number of complex care tasks the caregiver performs, mean ± SD	5.4 ± 3.1	5.8 ± 2.1
Health literacy score, mean ± SD	17.0 ± 1.5	17.3 ± 1.0
Social support, mean ± SD	15.7 ± 5.4	14.9 ± 6.2

Abbreviations: GED: general education development; IIG: immediate intervention group; WLG: waitlist control group.

### Intervention Delivery

During the study period, 6 cohorts (4 English language and 2 Spanish language) were recruited with caregivers from each cohort randomly assigned to the IIG or the WLG, with group size ranging from 3 to 8 caregivers. Of the 50 caregivers enrolled in the study, 3 dropped out before attending any intervention sessions, 2 in the IIG, and 1 in the WLG (see Figure 1), while 86% attended at least 5 of the 6 sessions (84% in the IIG and 88% in the WLG). To ensure that caregivers received the complete content, an additional weekly session was offered where caregivers who missed the group session met with the facilitator to review the material for the session they missed, with 14% of participants attending at least 1 “make-up” session. Attrition at 24 weeks was high among both groups, but more so among the IIG. Several care recipients died between 12 and 24 weeks, with data collection censored following the death of the person living with dementia, partially accounting for the high attrition.

Table 3 shows scores on primary and secondary outcomes at baseline, 12, and 24 weeks. The primary outcome, a measure of caregiver self-efficacy, was comparable between the 2 groups at baseline with a mean (±SD) of 6.8 ± 1.5 for the IIG and 6.4 ± 1.4 for the WLG. Baseline scores on the other 2 measures of self-efficacy, confidence in providing complex care and the 4 sub-scales of the Caregiver Confidence in Medical Sign/Symptom Management, were also similar between groups. Overall, baseline scores on the 3 different measures of confidence demonstrated that, on average, caregivers were moderately confident in care provision, scoring in the upper range of the scales. Scores on the secondary outcome measures were, for the most part, similar between groups at baseline. We also asked caregivers to provide the number of hospital stays and emergency room visits for the person living with dementia during the previous 3 months, with their reports indicating that these events were prevalent.

**Table 3.** Primary and Secondary Outcome Variables by Intervention Group.

Variable	IIG			WLG		
	Baseline (n = 25)	12 weeks (n = 20)	24 weeks (n = 14)	Baseline (n = 25)	12 weeks (n = 24)	24 weeks (n = 21)
Primary outcome						
Caregiver self-efficacy, mean (SD)	6.8 (1.51)	8.0 (0.96)	7.9 (1.10)	6.4 (1.38)	6.6 (1.50)	7.6 (1.72)
Secondary outcomes						
Confidence providing complex care, mean (SD)	4.1 (0.62)	4.3 (0.41)	4.4 (0.37)	4.1 (0.62)	3.9 (0.66)	4.4 (0.38)
CCMS, mean (SD)						
Knowledge about signs/symptoms	3.4 (0.98)	4.0 (1.11)	4.2 (0.72)	3.5 (1.06)	3.6 (1.04)	4.3 (0.66)
Managing cognitive signs/symptoms	3.6 (1.04)	4.1 (0.72)	4.2 (0.84)	3.8 (0.87)	3.7 (0.92)	4.3 (0.57)
Managing medical signs/symptoms	3.9 (1.08)	4.3 (0.72)	4.2 (0.58)	3.9 (0.89)	4.1 (0.96)	4.4 (0.59)
General medical management/responsiveness	4.1 (0.84)	4.3 (0.63)	4.5 (0.55)	4.2 (0.72)	4.0 (0.71)	4.5 (0.39)
Global health, mean (SD)						
Physical health	14.6 (2.6)	15.0 (2.15)	15.6 (2.98)	14.4 (2.86)	14.5 (2.80)	15.6 (2.78)
Mental health	13.4 (3.6)	13.6 (3.2)	13.6 (3.6)	13.3 (3.0)	12.6 (3.5)	14.6 (3.1)
Memory and behaviors checklist, mean (SD)						
Total score	10.8 (3.7)	11.4 (3.8)	6.1 (6.4)	10.4 (5.1)	10.7 (4.8)	8.2 (6.1)
Memory	5.4 (1.63)	5.6 (2.06)	3.2 (3.2)	5.3 (1.86)	5.1 (1.35)	4.1 (2.52)
Depression	3.0 (2.15)	3.4 (2.01)	1.8 (2.26)	3.1 (2.45)	3.0 (2.24)	2.1 (2.49)
Disruption	2.4 (1.66)	2.4 (1.63)	1.2 (1.57)	2.1 (1.91)	2.6 (2.34)	2.0 (2.17)
Caregiver appraisal of behaviors, mean (SD)						
Total score	15 (14.0)	19 (12.0)	14 (9.0)	17 (15.0)	15 (15.0)	17 (14.0)
Memory	5.8 (6.6)	8.3 (5.6)	5.7 (4.6)	4.0 (5.7)	3.2 (3.5)	4.3 (5.8)
Depression	4.6 (4.5)	6.5 (5.4)	4.6 (4.4)	7.0 (8.0)	6.0 (7.0)	6.0 (8.0)
Disruption	4.0 (4.4)	4.7 (5.2)	4.0 (4.0)	3.8 (4.4)	4.6 (5.2)	4.5 (4.0)
Hospitalizations in past 3 months, n (%)	6 (24)	3 (15)	0 (0)	4 (16)	5 (21)	2 (10)
ER visits in past 3 months, n (%)	10 (40)	7 (35)	1 (7)	4 (16)	6 (25)	2 (10)

Abbreviations: CCMS: caregiver confidence in medical sign/symptom management; ER: emergency room; IIG: immediate intervention group; WLG: waitlist control group.

### Impact of Aprendiendo Juntos/Learning Together Intervention on Caregiver Confidence

As seen in Table 4, there were consistent significant increases in caregiver confidence across confidence outcomes. We denote the mean and standard deviation of the change score by MC (mean of the change score) and SC (standard deviation of the change score). Caregiver self-efficacy significantly increased in the IIG (MC = 1.14, SC = 1.34) compared with the WLG (MC = 0.34, SC = 1.16) (*P* = .042). The difference between groups was clinically significant as evidenced by a moderate effect size (*d* = 0.64). Caregiver confidence for providing complex care, as measured with the Complex Care Task Scale, significantly increased in the IIG (MC = 0.28, SC = 0.49) compared with the WLG (MC = −0.17, SC = 0.40) (*P* = .002), showing a strong effect size (*d* = 1.00). Of the 4 sub-scales in the Caregiver Confidence in Medical Sign/Symptom Management questionnaire, there were significant increases in the IIG compared with the WLG in 2 subscales, the managing cognitive signs/symptoms (*P* = .004) and general medical management/ responsiveness (*P* = .003) sub-scales. No differences by group were observed in the remaining 2 sub-scales or in the other secondary outcomes: caregiver global health (physical and mental subscales) and the caregiver appraisal of behaviors. A series of multivariable linear regression models were used to examine the impact of the intervention on caregiver confidence, adjusting for baseline differences in language of intervention (English vs Spanish), employment status, and time caregiving. As seen in Table 5, results were similar to those found with the *t*-tests.

**Table 4.** Change From Baseline to 12 weeks, Immediate Intervention Group Versus Waitlist Control Group.

Variable	IIG	WLG	Between-group difference in change from BL		
	Change from BL mean (SD)	Change from BL mean (SD)	Mean (SD)	Effect size, *d*	*P*
Caregiver self-efficacy^a^	1.14 (1.34)	0.34 (1.16)	0.81 (1.25)	0.64	.04
Complex care task scale	0.28 (0.49)	−0.17 (0.40)	0.45 (0.45)	1.00	.002
CCMS					
Knowledge about signs/symptoms	0.65 (1.70)	0.07 (0.75)	0.58 (1.31)	0.45	.17
Managing cognitive signs/symptoms	0.63 (0.93)	−0.17 (0.74)	0.80 (0.84)	0.95	.004
Managing medical signs/symptoms	0.53 (0.99)	0.09 (0.81)	0.43 (0.91)	0.48	.13
General medical management/responsiveness	0.40 (0.79)	−0.23 (0.44)	0.63 (0.64)	0.99	.003
Global health					
Physical health	−0.20 (2.65)	0.18 (3.00)	−0.37 (2.83)	0.13	.67
Mental health	0.15 (2.85)	−0.50 (2.65)	0.65 (2.76)	0.24	.44
Memory and behaviors checklist					
Total score	1.10 (3.77)	0.33 (2.57)	0.77	0.24	.45
Memory	−0.05 (1.91)	−0.17 (1.58)	0.12 (1.75)	0.07	.83
Depression	0.90 (1.65)	−0.04 (1.43)	0.94 (1.54)	0.61	.05
Disruption	0.25 (1.86)	0.54 (1.44)	−0.29 (1.67)	0.18	.57
Caregiver appraisal of behaviors					
Total score	3.31 (11.6)	−0.40 (13.40)	3.71 (12.51)	0.30	.44
Memory	1.00 (5.95)	−0.61 (4.18)	1.61 (5.18)	0.31	.33
Depression	1.88 (5.57)	−0.58 (5.75)	2.45 (5.66)	0.43	.21
Disruption	0.19 (3.54)	1.18 (4.60)	−0.99 (4.11)	0.24	.49

Abbreviations: BL: baseline; CCMS: caregiver confidence in medical sign/symptom management; IIG: immediate intervention group; WLG: waitlist control group.

a Primary outcome.

**Table 5.** Multivariable Linear Regression Models for Caregiver Confidence Outcomes.

Outcome variable	Intervention status^a^	Beta (95% CI)	*P*
Caregiver self-efficacy	WLG	Reference	
	IIG	1.0 (0.19, 1.8)	.02
Complex care task scale	WLG	Reference	
	IIG	0.46 (0.17, 0.75)	.002
CCMS: knowledge about signs/symptoms	WLG	Reference	
	IIG	0.66 (-0.12, 1.4)	.09
CCMS: managing cognitive signs/symptoms	WLG	Reference	
	IIG	0.89 (0.35, 1.4)	.002
CCMS: managing medical signs/symptoms	WLG	Reference	
	IIG	0.52 (-0.07, 1.1)	.08
CCMS: general medical management/responsiveness	WLG	Reference	
	IIG	0.69 (0.27, 1.1)	.002

Abbreviations: CCMS: caregiver confidence in medical sign/symptom management; IIG: immediate intervention group; WLG: waitlist control group.

a Adjusted for employment status (employed vs unemployed), years caregiving (≤5 years vs >5 years), and language of intervention (Spanish vs English).

### Sustainability of Intervention Effects

To examine the sustainability of the intervention on caregiver confidence, data from the IIG only were used. Table 6 shows the changes in caregiver confidence assessed with the 3 different measures of confidence between baseline and 12 weeks, baseline and 24 weeks, and 12 and 24 weeks. Among caregivers in the IIG, caregiver self-efficacy, the primary outcome, increased significantly between baseline and 12 weeks (MC = 1.14, SC = 1.343; *P* = .001) and between baseline and 24 weeks (MC = 0.96, SC = 1.50; *P* = .033). Caregiver self-efficacy did not change between 12 and 24 weeks (MC = −0.23, SC = 1.352; *P* = .532), suggesting the increases seen at 12 weeks were sustained through 24 weeks. Similar patterns were observed among the other measures of caregiver confidence.

**Table 6.** Examination of Sustainability of Caregiver Confidence Among IIG Caregivers.

Variable	12 weeks–baseline			24 weeks–baseline			24–12 Weeks		
	Mean change	SD	*P*	Mean change	SD	*P*	Mean change	SD	*P*
Caregiver self-efficacy	1.14	1.343	.001	0.96	1.50	.03	−0.23	1.352	.53
Complex care task scale	0.28	0.494	.20	0.27	0.351	.013	−0.01	0.382	.93
CCMS									
Knowledge about signs/symptoms	0.65	1.696	.10	0.91	1.262	.02	0.20	1.44	.56
Managing cognitive signs/symptoms	0.63	0.934	.007	0.56	0.712	.01	−0.048	0.583	.77
Managing medical signs/symptoms	0.53	0.993	.03	0.36	0.929	.17	−0.20	0.59	.24
General medical management/responsiveness	0.40	0.791	.04	0.54	0.524	.002	0.10	0.664	.58

Abbreviations: IIG: immediate intervention group; CCMS: caregiver confidence in medical sign/symptom management.

## Discussion

In this longitudinal, randomized, waitlist controlled trial, the Aprendiendo Juntos/Learning Together intervention significantly improved caregiver self-efficacy in providing dementia care compared with a WLG. Notably, this significant improvement in confidence was consistent across the different measures of confidence utilized in this study, controlling for baseline group differences in employment status, years caregiving, and language of intervention, except for 2 sub-scales of the Caregiver Confidence in Medical Sign/Symptom Management. Changes were also clinically important as demonstrated by medium to large effect sizes. Examining change over time in the IIG showed that the results were sustained at 24 weeks (18 weeks after completing the intervention). The results did not show improvements in caregiver global health or in their appraisal of the care recipient’s behaviors.

Self-efficacy was the targeted outcome as it has been shown to be a modifiable attribute which can be learned and enhanced.^28^ Elements of the intervention aimed at building self-efficacy included practice exercises designed to facilitate mastery, coaching, and feedback from group facilitators, as well as caregivers modeling success for different aspects of complex care. Two subscales of the Caregiver Confidence in Medical Sign/Symptom Management did not show significant changes although they did demonstrate small effect sizes. These subscales contained items assessing knowledge of, and confidence in, managing hypertension and diabetes. The intervention provided limited content on these topics, with the subsequent scores demonstrating lower self-efficacy in managing these co-morbid conditions.

Previous research has demonstrated a relationship between caregiver self-efficacy and caregiver outcomes including burden, depression, and health-related quality of life.^10,13,29^ Given the significant increase in caregiver confidence following participation in the intervention, the lack of effect on caregiver self-rated mental health was unexpected. The average raw score on the mental health sub-scale of the Global Health measure was 13.5, equivalent to a *T*-score of 46 (population mean = 50). This score is similar to that reported by caregivers whose care recipients were in palliative care^30^ and also caregivers of recipients with heart failure.^31^ Dionne-Odom et al delivered a 4-week psychosocial and problem-solving support telephonic sessions facilitated by a trained nurse coach to heart failure caregivers and similarly reported no intervention effect on caregiver self-reported mental health.^31^ Chenoweth et al, in a pre-post design, compared individualized coaching to group coaching to usual care and reported non-significant improvements in caregiver health after the coaching.^32^ A possible explanation for the lack of intervention effects on mental health outcomes could be that, in addition to the underpowered sample size for these variables, the caregivers, on average, were not experiencing poor mental health, and thus there was no room for improvement. About 60% of caregivers reported providing care for more than 3 years, and it may be that they are more resilient over time.

The current study was designed to increase caregivers’ self-efficacy for complex care. It is important to consider the study findings more specifically within the context of the Latino culture. A rigorous process was followed in culturally tailoring the original LST intervention, including a community-engaged approach, with the Latino Family Caregiver Council guiding the process. In delivering the intervention, technology was offered to caregivers who did not have access to a computer or tablet, and support was offered to enhance caregiver comfort in using the videoconferencing software. The intervention was provided in both Spanish and English languages. There is likely to be, however, aspects of the Latino culture and belief system that were insufficiently addressed in the current study. Beliefs about their responsibility to family and caregiving may influence their caregiving experience, caregiver burden, and their willingness to seek support.^33^ Although there was content devoted to caregiver self-care, including a focus on emotional health, the dose may have been insufficient to further impact their mental health. A sufficiently powered study using mixed methods could further examine the intervention effects and improve our understanding of the content that is necessary and sufficient to build caregiver emotional health along with self-efficacy.

Latino caregivers are reported to be younger than caregivers of other racial and ethnic groups, with a higher proportion of adult children caring for a parent compared with other racial and ethnic groups.^34^ This is consistent with our caregiver participants, with an average age of 59 years and over 70% caring for a parent living with dementia. These demographic differences highlight the unique needs that may be faced by Latino family caregivers who are often providing care within the context of work and family responsibilities. In the current study, over 60% of caregivers reported changing their employment status to provide care. Furthermore, the main reasons for the refusal to participate in the study were employment and insufficient time for participation. These findings underscore the need not only for training resources that address their specific needs in terms of content and language but also for flexibility in the delivery. The need for flexibility in delivery was addressed in a limited fashion by offering the intervention at different times to better accommodate work schedules, and also through offering the weekly “make-up” session.

There is emerging literature on the benefits to family caregivers of technology-delivered interventions, including education, psychological/cognitive behavioral therapy, clinical care, and social support.^35^ Yet there is still a digital divide that affects caregivers of minority groups and underserved communities. In a nationally representative cross-sectional survey on caregivers’ perceptions of telehealth communication during the COVID-19 pandemic, Latino and Black/African American caregivers were more likely to express a desire to return to in-person communication compared with White caregivers.^36^ The Aprendiendo Juntos/Learning Together intervention was adapted from LST based on the expertise of the Latino Family Caregiver Council and semi-structured interviews with 14 Latino family caregivers. Based on participation rates, our study demonstrated that enrolled caregivers readily engaged with the digital conferencing platform, with a small percentage of caregivers requiring additional support through the provision of a tablet and technological support using the technology. It is, however, important to consider further enhancements to deliver the intervention using a combination of synchronized and asynchronous approaches for more equitable access.

### Limitations

Although recruitment for the sample included both community and clinical-based sources, caregivers who agreed to participate may not be representative of Latino family caregivers in general. The majority of the group had access to the technology needed for the study, and most were comfortable using it without additional training. Furthermore, those who agreed to participate had sufficient time, support, and motivation to attend a 6-week intervention program. While this selection bias may not affect the internal validity of the results, it may impact the generalizability of the findings to the broader population of Latino family caregivers.

This was a group-based intervention where caregivers learn from one another as well as from the intervention content. Providing a weekly make-up session was important to ensure that caregivers received the intervention content, with an opportunity for discussion with the facilitator as well as other caregivers who may have been attending the make-up session. This may, however, have changed the group dynamics when caregivers were missing from the larger cohort and receiving parts of the intervention in a smaller group.

There was a 12% attrition rate at the 12-week follow-up, higher in the IIG (n = 5) compared with WLG (n = 1). In examining the baseline data, there were some differences between the group of 6 caregivers compared with the remaining participants including a slightly younger average age, a higher percentage caring for a parent, and providing care for a shorter period of time. It is thus possible that the missing data at 12 weeks could have biased the estimate of the differences in confidence between groups, with results requiring further confirmation.

## Conclusions

Approximately 13% of Latinos aged 65 years or over live with Alzheimer’s disease or another form of dementia.^1^ To this growing group of Latino persons with some form of dementia, Latino families, in contrast to non-Latino families, are more likely to rely on family-based care compared with formal care services.^1^ There is an urgent need to increase the availability and accessibility of evidence-based interventions around care provision to benefit the health of these Latino family caregivers. The current study findings support the efficacy of the Aprendiendo Juntos/Learning Together intervention in increasing self-efficacy among Latino caregivers in providing complex care to their family members with dementia. The next step is to conduct a sufficiently powered RCT or a hybrid effectiveness-implementation trial. Future research should focus on how best to adapt the intervention for widespread dissemination among underserved Latino family caregivers and also examine mechanisms to better support their mental health through improved self-efficacy.
